# Agency and Communion in Brief Entire Life Narratives Across the Life Span

**DOI:** 10.1111/jopy.12990

**Published:** 2024-11-09

**Authors:** Nina F. Kemper, Theresa Martin, Lea Cohrs, Florian Schmiedek, Tilmann Habermas

**Affiliations:** ^1^ Department of Psychology Goethe University Frankfurt Frankfurt Germany; ^2^ DIPF, Leibniz Institute for Research and Information in Education Frankfurt Germany; ^3^ International Psychoanalytic University Berlin Berlin Germany

**Keywords:** agency, autobiographical memory, communion, life span development, life story, motivational themes, rank‐order stability

## Abstract

**Objective:**

The evolving life story is integral to personality, and motivational themes are central features of the life story. Personality implies individual differences that are relatively stable over time, but still allow for developmental processes. This study explored both long‐term stability and developmental change in thematic content of the life story.

**Method:**

Fulfilled and unfulfilled agency and communion were studied in brief entire life narratives across 4 measurements in 12 years in a cohort‐sequential design including six cohorts (*n* = 172; age 8–77).

**Results:**

Fulfilled agency and communion, as well as unfulfilled agency exhibited moderate rank order stability over 4 and 8 years, fulfilled communion showed even a modest 12‐year stability, whereas unfulfilled communion displayed an unsystematic pattern. Developmentally, multilevel analyses revealed an inverted U‐shaped association between age and both fulfilled and unfulfilled agency, peaking in mid‐life. Fulfilled communion declined after emerging adulthood, but unexpectedly did not increase again in old age. Unfulfilled communion showed no systematic age trends. Girls and women told life narratives with more fulfilled and unfulfilled communion, whereas genders did not differ in either kind of agency.

**Conclusion:**

The content of the life story exhibits rank‐order stability over time and systematic mean‐level development across the life span.

When Yasemin was asked to tell her life at age 20, she started with how she had come to Germany with her family when she was a child. In Turkey, she had lived with many relatives in a small village. In the new German city, she felt isolated and unrecognized. She was either not noticed or bullied by her classmates. But at some point she decided to stop being the victim. She talked back, hit back. She changed her look and became an outgoing person. After she had switched to a new school, she made friends and graduated successfully. At the end of her narrative, she stated how proud she was of what she had accomplished.

Yasemin narrated herself as someone who is capable of taking action and who has an impact on the course of their experiences. Her life narrative is an agentic narrative. *Agency* captures a basic human tendency towards self‐assertion and separation from others (Bakan 1966). Yasemin's story begins with a theme of unfulfilled agency, being a powerless victim of others' actions, and then introduces agentic moments of self‐mastery, expressed in the decision to actively change her life, and of achievement (see McAdams et al. 1996). Likewise, she overcomes the initial social isolation, a theme of unfulfilled communion, by connecting with others and feeling accepted by her classmates, expressing themes of communion. *Communion* captures the tendency to cooperate, closely relate to and merge with others (Bakan 1966). In this article, we study the relative stability of agency and communion in life narratives and how they vary with age. We contribute to the literature by extending the time interval (4, 8, and 12 years) and the age span (8–77) studied. In examining brief entire life narratives instead of single life story episodes, we aimed to support previous findings with a different method.

Trait theorists like Wiggins (1991) define agency and communion as basic dimensions of personality, with people showing different patterns of agentic (e.g., dominant, persuasive, ambitious) and communal (e.g., cooperative, loyal, sociable) traits. McAdams (1982) transferred this idea to another level of personality, the life story. The life story is an internalized and developing narrative of the self (McAdams 1993) that integrates change in life circumstances and personality across a lifetime (Habermas and Bluck 2000).

Life narratives are considerably stable (Köber and Habermas 2017; Peters et al. 2023). However, findings on the stability of themes of agency and communion in life stories are less clear. The two only studies of stability of agency and communion in life stories found either moderate rank‐order stability of agency, but not of communion across 3 years (McAdams et al. 2006) or a moderate stability of communion, but not of agency across 6 years (Sengsavang et al. 2017). Both studies averaged scores across several narratives of specific events such as turning points, but not of entire life narratives.

The process of identity development uses the life story format from adolescence onwards (Erikson 1950, 1968). The life story changes in at least two ways. First, as life is being lived forwards, it needs to be updated by adding new parts of life. Second, and more important for our purpose, individuals' present motives and goals influence which memories are selected to be part of the life story in a particular phase of life (McAdams et al. 1996; Woike 2008) and color the content of autobiographical memories (Conway and Pleydell‐Pearce 2000). Accordingly, two studies demonstrated that most accessible autobiographical memories from varying phases of life tended to correspond to the Eriksonian themes dominant in each of the respective life phases (Conway and Holmes 2004; Nusser, Zimprich, and Wolf 2023). Therefore, we expect life span theories of social motivation to allow predicting also the age‐specific salience of agency and communion in life narratives. We first summarize theories on the trajectories of agency and communion across the life span, and then report findings on mean‐level change of agency and communion as features of the life story.

In Erikson's (1950, 1968) theory, the developing individual must pass through eight stages across the life course. These stages are influenced both by biological maturation and sociocultural conventions and norms, as are Havighurst' (1972) developmental tasks. Both in turn are influenced by the macro‐social systems of work and education (Hagestad and Dykstra 2016). Each stage involves a distinctive crisis associated with a cluster of typical motives (Conway and Holmes 2004). Motives may be classified as more agentic or more communal.

Agentic motives become increasingly important during adolescence. Adolescents aim to gain autonomy from their parents and to actively explore who they are and want to become. In middle adulthood, generative aims of passing on knowledge, taking on responsibility for others, or improving others' lives are highly agentic. Later in life, when the task is to come to terms with the life that has been lived and with impending death (*integrity*; Erikson 1968), self‐assertion and separation from others are no longer central. We thus summarize that Erikson's theory predicts agentic striving to follow an inverted U shape, with a peak in midlife, and a subsequent decline. This fits well with age trajectories of the capacity to exert agency predicted by the motivational theory of lifespan development (Heckhausen and Schulz 1995; Heckhausen 2018) and with survey data finding a peak of agency in mid‐adulthood (Mirowsky and Ross 2007).

Communion also increases in importance in the adolescent years. This is the time of “best friends” and of first romantic relationships. In young adulthood, the general need for communion transforms into a more focused need for intimacy, which involves accepting differences, resolving conflicts, and honoring commitments (Bar‐Yam Hassan and Bar‐Yam 1987; Erikson 1968). Concomitantly, people become more socially selective, a trend that intensifies in middle adulthood. In old age, as the future is perceived as more limited, life centers more on emotional needs (Carstensen, Isaacowitz, and Charles 1999; Erikson 1968). This brings relationships back into focus, the striving for communion increases again. Thus, communion motivation appears to peak in adolescence, subsequently decline throughout early and middle adulthood, and to rebound in old age.

We expect these trajectories of agency and communion motivation to be reflected in how life stories change with age. To date, findings show no clear pattern. As for agency, McAdams et al. (2006) found no mean‐level change in emerging adulthood (age 18–23), nor did Sengsavang et al. (2017) in young adulthood (age 26–32). However, the age spans studied covered only one life phase. Cross‐sectionally, agency in life story narratives was positively associated with age both in a sample of adolescents (McCabe and Dinh 2016) and of middle‐aged adults (McAdams et al. 2004). One study covering adulthood (15–69 years) cross‐sectionally found the expected curvilinear relation with age with a peak in midlife (30s) (Buehler, Weidmann, and Grob 2021), whereas another found no linear relation across adulthood (22–72 years) and did not report on a curvilinear relationship (McAdams et al. 1996).

Communion did not change longitudinally between ages 18 and 23 (McAdams et al. 2006), and between ages 26 and 32 it only increased in participants who had become parents (Sengsavang et al. 2017). Cross‐sectionally, one study found a decrease between emerging and middle‐adulthood (McAdams et al. 2004), one found the trend of an increase between ages 15 and 69 (Buehler, Weidmann, and Grob 2021), and two others did not reveal any age‐differences in adolescence (McCabe and Dinh 2016) and between ages 22 and 72 (McAdams et al. 1996). Thus, although there is some weak support for an inverse curvilinear change of agency with age, findings for communion are contradictory.

Because McAdams' original coding scheme, which we used for this study, only captures fulfilled motives, it might miss that in certain life phases agentic or communal needs are present but unfulfilled. Adler et al. (2012) introduced this distinction in a study of personality disorders, which has since been used in a series of studies of clinical groups by Thomsen and her group (e.g., Holm, Thomsen, and Bliksted 2018; Jensen et al. 2021). When Yasemin talked about taking action, she also talked about being powerless and at the mercy of circumstances. Her narrative is not only about belonging but also about being an outsider. When Yasemin told her life repeatedly across many years, the theme of not belonging varied, but remained throughout her retellings. In other participants' repeated life narratives, we observed unfulfilled themes to pop up and disappear again. To date, neither the stability of unfulfilled themes of agency and communion nor their variation with age have been studied.

Finally, the literature has consistently suggested (Chodorow 1978; Gilligan 1982) that for women themes of communion are more important than for men and that women develop a more interconnected sense of self, whereas men's sense of self stresses personal achievement and autonomy. These gender differences appear to be largely due to the socialization of gender norms and to gender‐based division of labor and distribution of power in Western societies (e.g., Wood and Eagly 2012). Although self‐report measures consistently find more agentic self‐descriptions by men than by women (Diehl, Owen, and Youngblade 2004; Hsu et al. 2021; Ross and Mirowsky 2002), these expectations are only partially reflected in themes of autobiographical narratives. Young women's life story memories (aged 18–23) contained more communion than young men's, but not less agency (Grysman et al. 2016; McAdams et al. 2004, 2006), while in older samples genders did not differ in communion either (McAdams et al. 1996, 2004). However, there were no gender differences in the relational content of adolescents' turning point memories, possibly because communion is similarly salient for boys and girls in this life phase (McLean and Breen 2009).

The present study set out to track the stability of themes of agency and communion in life narratives across many years to study how their presence varies with time and age, both in terms of fulfilled and of unfulfilled agency and communion. We expected both themes to exhibit at least modest rank‐order stability over 4 years, then decreasing moderately across the following 8 years. For mean‐levels of agency, we expected an inverted U curve with a peak in midlife, and for communion a cubic trajectory with a relative peak in adolescence and subsequent decline throughout early and middle adulthood and a rebound in old age. We also explored rank‐order stability and mean‐level change of unfulfilled agency and communion as well as gender differences. Finally, we took a closer look at the narratives to explore what it means when agency and communion change. How is agency and communion differently expressed in life narratives of people of different ages? Guided by this question, we attempted to deepen the understanding of our quantitative results.

## 1 Methods

### 1.1 Participants

The present data comprise four waves of the longitudinal MainLife Study (Habermas 2022), with measurements in 2003, 2007, 2011, and 2015, originally designed to test the development of the life story. The initial sample consisted of *N* = 114 participants, with about equal numbers from four cohorts aged 8, 12, 16, and 20 years. Four years later, a total of 104 subjects participated again, 99 participated a third, and 87 participated a fourth time (dropout rates: 8.8%, 4.8%, and 12.1%, respectively). In 2007, two groups aged about 40 and 65 years (*N* = 58) were added to test life span development. Of these, 51 individuals participated again in 2011 and 48 again in 2015 (dropout rates: 12.1% and 5.9%, respectively). Gender was about equally distributed in the six cohorts (see Table 1), and dropouts did not differ with regard to age, gender, and education (see Table S1).

**TABLE 1:** Age of participants in years (mean, standard deviation), number of participants, and gender distribution.

	Cohort 1	Cohort 2	Cohort 3	Cohort 4	Cohort 5	Cohort 6	*n*
Wave 1	8.58 (0.27)	12.41 (0.37)	16.53 (0.44)	20.50 (0.57)			
*n*	27	31	28	28			114
Females	13	17	13	15			
Wave 2	12.90 (0.52)	16.58 (0.42)	20.70 (0.51)	24.93 (0.73)	41.39 (2.86)	65.38 (2.73)	
*n*	23	29	26	26	28	30	162
Females	12	16	12	14	14	15	
Wave 3	17.03 (0.48)	20.58 (0.39)	24.61 (0.41)	28.90 (0.67)	45.08 (3.02)	68.73 (2.65)	
*n*	23	27	26	23	22	29	150
Females	13	15	11	13	11	15	
Wave 4	21.31 (0.61)	25.07 (0.43)	29.20 (0.65)	33.49 (0.77)	49.44 (2.89)	73.34 (2.56)	
*n*	24	22	20	21	20	28	135
Females	13	13	11	11	9	14	
Total							561

In 2003, the youngest cohort was the higher achieving half of third graders from an elementary school, whereas Cohorts 2, 3, and 4 were present or former students of a German higher‐track high school (Gymnasium). Its mixed social composition, mainly middle class with a substantial proportion of lower‐class background, was comparable to that of the elementary school population. Cohorts 5 and 6 were recruited via flyers and among continuing education university students.

Participants were recompensed with €20 in 2003, €40 in 2007 and 2011, and with €60 in 2015. At each follow‐up, we contacted participants up to three times by letter, then via email, phone, and social media and obtained parental informed consent for minors.

### 1.2 Procedure

In 2003, Cohorts 1 to 4 were tested twice, 2 weeks apart, by two different female interviewers. At the other waves, all participants were tested only once by other female interviewers, unknown to the participants. This served to ensure that participants did not simply update their last telling but told the entire life anew. Across measurement times participants in the four younger cohorts provided up to five entire life narratives, the two older cohorts provided up to three entire life narratives. In total, 666 life narratives were collected, of which 561 remained in the present sample after excluding the second measurement from Wave 1. Most participants came to the lab for the interview. The three younger cohorts of Wave 1 were interviewed at school, and some participants were visited at home if necessary.

Participants wrote their seven most important memories on index cards and put them in chronological order on the table in front of them. Then participants were instructed to narrate their lives for about 15–20 min so as to explain how they had become the person they were at present. They were asked to include the seven most important memories. Interviewers only encouraged to continue but asked no questions (for instructions, see Habermas and de Silveira 2008).

Life narratives were tape‐recorded, transcribed verbatim, and divided into propositions, that is, main or subordinate clauses (cf. Habermas and de Silveira 2008). In contrast to more interactive interview techniques asking for narratives of selected episodes (e.g., Guided Autobiography; McAdams 1997), we did not intervene in the narratives, and we asked for entire life narratives that encompass the narrator's development from the beginning to the present day. To code themes similarly as McAdams codes narratives of single life story episodes, we divided life narratives into thematic segments typically containing a specific, datable event, and in some cases recurrent events, descriptions, or evaluative summaries (cf. Habermas and Diel 2013).

### 1.3 Coding Agency and Communion

McAdams' (2001) manual contains four subthemes each for agency and communion (see Tables S2 and S3). Motivational themes are only coded if they are expressed in positive terms, that is, if the protagonist could satisfy their agentic or communal need. For example, the description of the self‐mastery theme states: “the story protagonist strives *successfully* to master, control, enlarge, or perfect the self.” To complement these codes, we defined unfulfilled equivalents for each of the eight original subthemes as occasions when a need becomes thematic but remains unfulfilled. The unfulfilled equivalent for self‐mastery was: “The story protagonist strives *unsuccessfully* to master, control, enlarge, or perfect the self.” In both cases, the pursuit of self‐enlargement and development is thematic, but the difference is whether or not it is realized. Coders assessed how many fulfilled or unfulfilled subthemes per agency or communion were present in each segment, thus providing a total of four scores ranging from 0 to 4 for each segment following McAdams’ manual.

A total of four independent coders, two for fulfilled and unfulfilled agency and two for fulfilled and unfulfilled communion, were trained by the first author. Interrater reliabilities were then calculated on the basis of 20 consecutively coded life narratives. Interrater reliabilities for fulfilled and unfulfilled agency subthemes ranged from Cohen's *κ* = 0.79 to *κ* = 1.00. Unfulfilled empowerment was never coded. Interrater reliability for fulfilled and unfulfilled communion subthemes ranged from *κ* = 0.86 to *κ* = 1.00. Then each coder coded half of the remaining narratives. To check for potential coder drift, we calculated an additional control reliability based on another 12 narratives, which were interspersed randomly among the remaining narratives unknown to the coders. Control interrater reliability for agency ranged from *κ* = 0.72 to *κ* = 1.00. Neither of the two empowerment themes was coded. Control interrater reliability for communion ranged from *κ* = 0.87 to *κ* = 1.00. Dialogue was only coded once, and unfulfilled dialogue was never coded. Disagreements regarding the narratives used to measure interrater reliability were resolved by discussion.

Scores for fulfilled and for unfulfilled agency and communion respectively were summed across the segments of each life narrative, divided by the total number of segments, and multiplied by 100. The resulting four scores of a life narrative thus indicate the average presence of fulfilled or unfulfilled agency or communion subthemes in segments.

### 1.4 Data Analysis Strategy

Rank‐order stabilities of fulfilled and unfulfilled agency and communion were measured using retest‐correlations. To investigate age trends, we applied mixed models for repeated measures, using maximum likelihood estimation in RStudio Version 4.1.0, procedure lmer in the lme4 package (Bates et al. 2015). First, we divided all scores by 10 to avoid very small values for the unstandardized regression coefficient of the quadratic predictor. Then, we estimated different models for each of the four motivational themes, comparing model deviances with *χ* ^2^ tests, to find the best fitting one. First, we entered gender as a fixed effect and compared it to the baseline (random intercept) model. Sequentially, we added fixed age, fixed quadratic age, and the interaction terms of gender with age and of gender with quadratic age. We refrained from testing the cubic effect for communion because the scatterplot clearly showed there was none. Then, we tested for random effects for each of the predictors, which did not improve the model fit significantly, so that we ended up with a random intercept, fixed slope model for four themes.

## 2 Results

### 2.1 Descriptive Statistics

Mean values and standard deviations are presented in Table 2. Overall, communion was more common in life narratives than agency. Both unfulfilled themes were considerably less frequent than their fulfilled counterparts. Concerning subthemes, love/friendship was by far the most frequent one, followed by unity/togetherness, self‐mastery, achievement/responsibility, and unfulfilled love/friendship.

**TABLE 2:** Means and standard deviations of relative frequencies of motivational themes (*N* = 561).

	Female	Male	Total
Agency	11.26 (9.68)	12.75 (10.99)	11.98 (10.35)
Self‐mastery	4.95 (6.46)	4.47 (6.84)	4.72 (6.64)
Status/victory	2.01 (3.73)	2.97 (5.19)	2.48 (4.52)
Achievement/responsibility	3.95 (5.28)	5.12 (5.94)	4.51 (5.64)
Empowerment	0.33 (1.34)	0.19 (0.99)	0.26 (1.18)
Communion	19.19 (13.70)	15.59 (12.14)	17.45 (13.08)
Love/friendship	11.74 (9.24)	9.15 (8.99)	10.49 (9.21)
Dialogue	0.56 (2.00)	0.46 (1.63)	0.51 (1.82)
Caring/help	1.96 (3.94)	1.28 (2.84)	1.63 (3.47)
Unity/togetherness	4.94 (6.00)	4.71 (6.07)	4.82 (6.03)
Unfulfilled Agency	7.06 (8.34)	5.75 (7.52)	6.42 (7.98)
Self‐mastery	3.68 (5.86)	2.63 (4.96)	3.17 (5.47)
Status/victory	1.84 (3.58)	1.39 (3.04)	1.62 (3.34)
Achievement/responsibility	1.53 (2.97)	1.73 (3.99)	1.63 (3.50)
Empowerment	0.00 (0.00)	0.00 (0.00)	0.00 (0.00)
Unfulfilled Communion	6.83 (7.42)	4.45 (6.33)	5.68 (7.01)
Love/friendship	4.51 (5.50)	3.43 (5.49)	3.99 (5.52)
Dialogue	0.29 (1.43)	0.11 (0.75)	0.20 (1.15)
Caring/help	0.50 (1.67)	0.36 (1.41)	0.43 (1.55)
Unity/togetherness	1.54 (3.33)	0.55 (2.08)	1.06 (2.84)

The relative frequencies of agency and communion did not correlate with each other (*r* = −0.04). We found a small association of agency with unfulfilled agency (*r* = 0.23, 95% CI [0.16, 0.30]) and of communion with unfulfilled communion (*r* = 0.26, 95% CI [0.19, 0.33]). There was a very small and negative relationship between communion and unfulfilled agency (*r* = −0.10, 95% CI [−0.17, −0.02]).

### 2.2 Stability of Individual Differences in Agency and Communion

All four themes exhibited medium‐sized to large rank‐order stability across 4 years (see Table 3). For the 4‐year intervals, the strength of the association increased with the age of the participants—only correlations between Waves 3 and 4 were large, those between earlier Waves of medium size. Agency, communion, and unfulfilled agency also showed medium‐sized stability across 8 years, and communion even exhibited medium‐sized stability across 12 years. For unfulfilled communion, no systematic pattern emerged. Unexpectedly, the relative stability of themes did not systematically decrease between 4, 8, and 12 years. To explore whether this might have been due to an increase of stability with age in the younger cohorts (Köber, Schmiedek, and Habermas 2015; Köber and Habermas 2017, due to an increase in coherence/stability of life narratives), which might have counteracted a decrease of stability with time, we visually inspected correlations calculated separately for three pairs of adjacent cohorts (see Table S4). However, correlations did not systematically decrease over time even if calculated separately for the oldest two cohorts for whom no developmental gains in stability could be expected.

**TABLE 3:** Spearman rank correlations across time for motivational themes, included cohorts, and number of participants.

	Agency	Communion	Unfulfilled Agency	Unfulfilled Communion	Included Cohorts	*n*
*Compared time interval*						
2 weeks						
Wave 1	0.46*	0.66*	0.52*	0.37*	1–4	105
4 years						
Wave 1–2	0.27*	0.35*	0.25*	0.03	1–4	104
Wave 2–3	0.32*	0.39*	0.20*	0.33*	1–6	145
Wave 3–4	0.41*	0.47*	0.45*	0.41*	1–6	129
8 years						
Wave 1–3	0.26*	0.24*	0.27*	0.19	1–4	99
Wave 2–4	0.27*	0.41*	0.25*	0.21*	1–6	131
12 years						
Wave 1–4	−0.07	0.34*	0.01	0.40*	1–4	87

* *p* < 0.05.

### 2.3 Change of Relative Frequencies of Themes With Age

**FIGURE 1:**
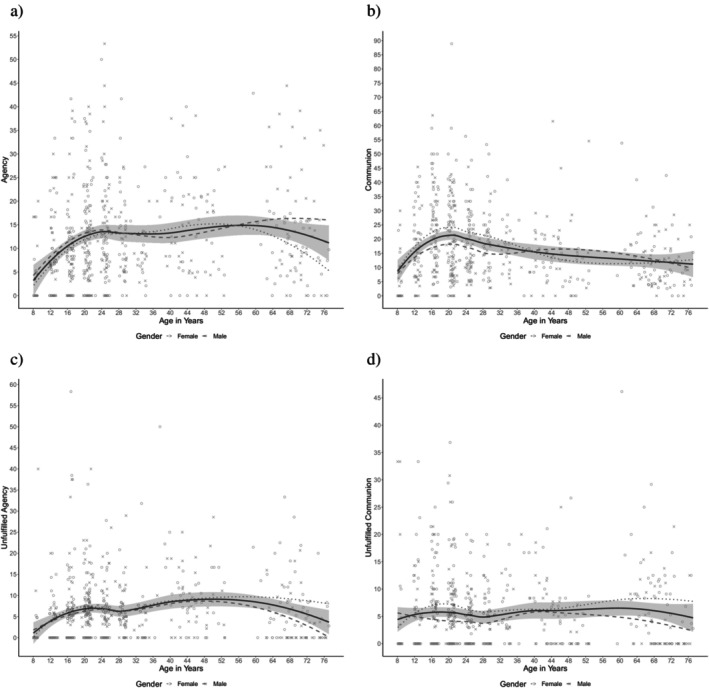
Association between age and themes of (a) fulfilled agency, (b) fulfilled communion, (c) unfulfilled agency, and (d) unfulfilled communion. Trend lines were included using the default settings of the loess method as implemented in the R package ggplot2 (Wickham 2016). The 95% confidence interval of the overall mean is displayed in gray.

The hypothesis that mean‐levels of fulfilled agency increase from childhood throughout adolescence and middle adulthood and decrease in older adulthood (negative quadratic distribution) was confirmed. The best model for age effects on agency included a random intercept at age 8, a fixed positive linear slope, and a fixed negative quadratic slope (Table 4). Figure 1a portrays the life span development of agency showing the expected inverted U shape. There was no effect of gender and no interaction of age or quadratic age with gender.

**TABLE 4:** Multilevel models for the effects of gender and age on fulfilled agency (*n* = 561).

	Baseline				Best Model			
	Estimator	SE	95% CI		Estimator	SE	95% CI	
			LL	UL			LL	UL
Fixed effects								
Intercept	12.07*	0.56	10.87	13.11	5.05*	1.32	2.47	7.76
Gender^a^					1.79	1.09	−0.10	3.95
Age^b^					0.53*	0.10	0.32	0.72
Age^2^					−0.01*	0.00	−0.01	−0.00

	Variance	SD	95% CI		Variance	SD	95% CI	
			LL	UL			LL	UL
Random effects								
Intercept	28.41	5.33	4.16	6.39	27.30	5.23	3.99	6.17
Residual	78.71	8.87	8.26	9.53	73.77	8.60	7.95	9.24
Model fit								
Deviance	4173				4139			
AIC	4179.2				4151.1			

*Note:* Sequentially adding predictors was tested with Δ*χ* ^2^ tests (df = 1) based on model deviance (−2Log‐Likelihood). 95% confidence intervals were based on bootstrapping.

Abbreviations: AIC = Akaike Information Criterion; I = confidence interval; LL = lower limit; UL = upper limit.

^a^ Gender was coded with 0 for female and 1 for male.

^b^ Age was entered centred at 8 years (according to the youngest age group).

* *p* < 0.05.

The hypothesis that mean levels of fulfilled communion first peak after adolescence and rise again in later adulthood (cubic distribution) was partially confirmed: Only the first peak could be identified (Figure 1b). The best model for age effects on communion included a random intercept at age 8, a fixed positive linear slope, and a fixed negative quadratic slope (Table 5). Additionally, women expressed more communion in their life narratives than men (Table 5, Figure 1b). Neither age nor quadratic age interacted with gender.

**TABLE 5:** Multilevel models for the effects of gender and age on fulfilled communion (*n* = 560).

	Baseline				Best Model			
	Estimator	SE	95% CI		Estimator	SE	95% CI	
			LL	UL			LL	UL
Fixed effects								
Intercept	17.31*	0.75	15.80	18.69	18.45 *	1.70	15.38	21.39
Gender^a^					−3.35*	1.44	−6.05	−0.46
Age^b^					0.24*	0.12	0.02	0.48
Age^2^					−0.01*	0.00	−0.01	−0.00

	Variance	SD	95% CI		Variance	SD	95% CI	
			LL	UL			LL	UL
Random effects								
Intercept	61.35	7.83	6.36	9.13	53.43	7.31	5.68	8.47
Residual	110.90	10.53	9.76	11.36	108.41	10.41	9.68	11.17
Model fit								
Deviance	4400				4375			
AIC	4406.4				4387.4			

*Note:* Sequentially adding predictors was tested with Δ*χ* ^2^ tests (df = 1) based on model deviance (−2Log‐Likelihood). 95% confidence intervals were based on bootstrapping.

Abbreviations: AIC = Akaike Information Criterion; CI = confidence interval; LL = lower limit; UL = upper limit.

^a^ Gender was coded with 0 for female and 1 for male.

^b^ Age was entered centred at 8 years (according to the youngest age group).

* *p* < 0.05.

Unfulfilled agency showed the same inverted U‐shaped distribution as fulfilled agency (Figure 1c). The best model for unfulfilled agency also included a random intercept at age 8, a fixed positive linear slope, and a fixed negative quadratic slope (Table 6). There were neither gender effects nor interaction effects. In contrast, unfulfilled communion did not vary with age (Table 7), but with gender. Across the life span, women expressed more unfulfilled communion than men did (Table 7, Figure 1d).

**TABLE 6:** Multilevel models for the effects of gender and age on unfulfilled agency (*n* = 561).

	Baseline				Best Model			
	Estimator	SE	95% CI		Estimator	SE	95% CI	
			LL	UL			LL	UL
Fixed effects								
Intercept	6.54*	0.43	5.66	7.37	4.02*	0.94	1.96	6.23
Gender^a^					−1.32	0.83	−2.85	0.24
Age^b^					0.29*	0.08	0.14	0.44
Age^2^					−0.00*	0.00	−0.01	−0.00

	Variance	SD	95% CI		Variance	SD	95% CI	
			LL	UL			LL	UL
Random effects								
Intercept	17.31	4.16	3.08	4.99	14.50	3.81	2.75	4.56
Residual	47.06	6.86	6.39	7.39	46.95	6.85	6.39	7.35
Model fit								
Deviance	3886				3870			
AIC	3892.4				3881.6			

*Note:* Sequentially adding predictors was tested with Δ*χ* ^2^ tests (df = 1) based on model deviance (−2Log‐Likelihood). 95% confidence intervals were based on bootstrapping.

Abbreviations: AIC = Akaike Information Criterion; CI = confidence interval; LL = lower limit; UL = upper limit.

^a^ Gender was coded with 0 for female and 1 for male.

^b^ Age was entered centred at 8 years (according to the youngest age group).

* *p* < 0.05.

**TABLE 7:** Multilevel models for the effects of gender and age on unfulfilled communion (*n* = 560).

	Baseline				Best Model			
	Estimator	SE	95% CI		Estimator	SE	95% CI	
			LL	UL			LL	UL
Fixed effects								
Intercept	5.67*	0.36	4.96	6.37	6.86*	0.48	5.90	7.72
Gender^a^					−2.44*	0.69	−3.72	−1.04
Age^b^								
Age^2^								

	Variance	SD	95% CI		Variance	SD	95% CI	
			LL	UL			LL	UL
Random effects								
Intercept	9.39	3.07	2.14	3.91	8.04	2.84	1.73	3.55
Residual	39.72	6.30	5.86	6.78	39.63	6.30	5.86	6.80
Model fit								
Deviance	3748				3736			
AIC	3767.0				3744.0			

*Note:* Sequentially adding predictors was tested with Δ*χ* ^2^ tests (df = 1) based on model deviance (−2Log‐Likelihood). 95% confidence intervals were based on bootstrapping.

Abbreviations: AIC = Akaike Information Criterion; CI = confidence interval; LL = lower limit; UL = upper limit.

^a^ Gender was coded with 0 for female and 1 for male.

^b^ Age was entered centred at 8 years (according to the youngest age group).

* *p* < 0.05.

### 2.4 Digging A Little Deeper Into the Narratives

We quantitatively analyzed the average of the sum of four agentic and communal subthemes respectively. To illustrate the heterogeneity within the two motivational themes and how they changed over the life span, we will take a deeper qualitative look at the narratives, presenting prototypical expressions for agency and communion by age. The youngest participants were 8‐year‐old third graders when they first told their lives. They narrated few agentic topics. Self‐mastery was never mentioned, but a few achievement and status codes revealed the relevance of social comparisons and competition at school. Playing and fighting with friends and being separated from friends or siblings were the communal topics at this early age.

In the following years, school became increasingly important. Getting good grades was a big issue and, correspondingly, failure in tests, often despite having worked hard for them. In the context of achievement, agency was often about failing, then trying again, or finding alternative ways. But school was also central as a context for self‐mastery. One theme was changing class or school after realizing that one does not feel comfortable with classmates or teachers. Sometimes the solution was to change oneself, especially one's body. Some of the self‐mastery moments included dyeing hair or getting smoother skin to feel more attractive and accepted. Another context for self‐development were sports clubs or political organizations, but also the family: One participant told how she had decided to become a better sister after having realized how much she had rejected her sibling. Communion was mostly about the ups and downs in the special relationship to a best same‐gender friend (chumship). Belonging and being excluded was also a prominent topic. In the transition from early to late adolescence, the first romantic and sexual relationships appeared on the scene, and the whole drama of falling in love, being rejected, trying again, getting intimate, and breaking up was told at length in the narratives.

In young adulthood, school years still played a role, but young adults also talked about experimenting with different careers and fields of study, about how they broadened their horizons through travels, stays abroad, or change of residence. Integrating such experiences into their life narratives they often used self‐mastery expressions, such as having felt more self‐confident after having studied abroad or chosen a major in College against their parents' objections. Achievement was also a frequent topic among young adults, talking about accomplishments in school, College, training, or in the first years of employment. Concerning communion, they spoke about their long‐term friendships as well as about having found new friends after moving to another city.

In the transition to middle adulthood, starting a family became an issue and earlier love relationships disappeared from the life story. A married 28‐year‐old participant reflected: “I left out my boyfriend from back then, because he's kind of not that important to me anymore.” Accordingly, how she met her now husband took up much space in her life narrative. Becoming a parent was described as an intense, often exceptionally gratifying experience, coded as caring and help. But young adults without children also expressed their concerns for others; one young woman narrated how she took great care of a colleague who had fallen into coma after an accident.

In midlife, themes of agency were about making important life decisions and handling crises, such as leaving a well‐paid job because it was not fulfilling. Unfulfilled agency concerned needs to adapt to unanticipated situations like chronic illness, often followed by a fulfilled self‐mastery expression after adjustment. Even in middle adulthood, people still talked about having felt isolated in adolescence. When it comes to being excluded and bullied at school, time seems not enough to heal wounds. On the other hand, the importance of long‐term relationships was often emphasized. In mid‐adulthood, participants did not talk about individual friends, but more generally mentioned that they still kept in touch with old friends. Positive feelings about finding a life partner and becoming parents was still an important communion theme.

Agency in old age consisted mainly of status and victory codes, especially for men. They had been born during or shortly after WW II and had been socialized during the economic boom in West‐Germany between 1950 and 1970. Older male participants tended to narrate successful careers and mentioned status objects like houses and cars. This contrasted with the negative expressions of self‐mastery of women in the same birth cohort. As one female participant summed it up: “If the man earns the money, then the woman has to take care of everything else, and I really realized this much too late.” Not having made the most of one's potential was a common unfulfilled self‐mastery theme of older women. Other expressions of unfulfilled agency that appeared in reviewing one's whole life were the struggle to create a home for oneself, and unresolved family conflicts. Communion themes regarded mostly children and grandchildren, but some older adults also talked about activities with friends or about their engagement in volunteer activities. Taken together expressions of agency and communion not only varied in frequency with age but also referred to different experiences at different stages of life. On the other hand, some topics remained throughout the years, especially experiences of social exclusion, but also the joy of becoming a parent.

## 3 Discussion

This longitudinal study of the development of motivational themes supports the idea that they are relatively stable and distinct features of the life story and that their mean presence changes systematically with age. Fulfilled agency and communion showed substantial rank‐order stability across 4 and 8 years, communion even across 12 years. Rank‐order stability of unfulfilled agency was comparable, but that of unfulfilled communion was more erratic across the different waves of the study.

Thematic content was more stable in this study than in the previous studies (McAdams et al. 2006; Sengsavang et al. 2017). This is probably due to methodological differences. Narrating an entire life coherently probably results in more stability than choosing several unrelated key point episodes and narrating them one by one, because well‐organized material is remembered better (Craik and Lockhart 1972). The more restrictive elicitation method of the present study probably activated a conscious top‐down search in the Self‐Memory‐System (Conway 2005; Conway, Justice, and D'Argembeau 2019; cf. Martin et al. 2023). This process tends to be less prone to situational influences and more influenced by the present self‐concept and the central characteristics of the stored life story schema (Bluck and Habermas 2000; Conway, Justice, and D'Argembeau 2019). A possible objection is that agency and communion are more stable in the present study because participants tended to re‐narrate the same events, since a complete life story usually follows a life script. Indeed, the percentage of re‐narrated events in the present MainLife study is high (as reported earlier, Köber and Habermas 2017) compared to other studies of thematic stability. However, narrating the same events does not necessarily result in stability of agency or communion, because we did not code the events themselves but the aspects that were highlighted when narrating them. For example, a marriage was coded for communion only if a warm, emotional quality could be discerned. So not only what people remember is relatively stable but also how they remember it (in a related vein, cf. Peters et al. 2023).

Surprisingly, stability did not decrease systematically after the first 4 years, and appeared to increase with age. The increase of stability with age in the younger cohorts did not explain the absence of a decrease in stability of themes with time. However, long‐term stability was always lower than stability across only 2 weeks. In contrast, McAdams et al. (2006) found a clear decrease in stability between 3 months and 3 years only for communion, whereas agency even showed a small increase. Taken together, the change of stability over time warrants further study.

Mean levels of agency increased up to mid‐adulthood and decreased thereafter as expected. Our expectation that communion peaks after adolescence, then decreases, and increases again in old age was only partially supported, because communion in fact did peak in early adulthood, but declined across the remaining life span. Unfulfilled agency showed the same inverted U‐distribution as fulfilled agency, although it was much less frequent. Unfulfilled communion showed no systematic age trends.

This study suggests that the life story changes over time in a way that is consistent with developmental theory. According to Erikson (1968), the question of identity becomes salient in adolescence, and this is also the time when the ability to construct a life story develops (Peters, Schmiedek, and Habermas 2024). Although two earlier studies found autobiographical memories to be colored by the theme dominant at the time of the remembered event (Conway and Holmes 2004; Nusser, Zimprich, and Wolf 2023), we demonstrated that entire life narratives are colored by the salience of motivational themes at the time of telling. The finding of a peak of agency in midlife is in line with previous research on age differences in motivational themes (Buehler, Weidmann, and Grob 2021; McAdams et al. 2004; McCabe and Dinh 2016), and with theories of agency motivation over the life span (Heckhausen and Schulz 1995).

The trajectory of frequencies of fulfilled communion mirror findings of studies on friendships and social networks over the life course, showing that the average number of friends increases during childhood and adolescence and diminishes with age in the adult years (Gillespie et al. 2015; Bruine de Bruin, Parker, and Strough 2020). However, the hypothesis that communion increases in older people because they focus more on emotionally fulfilling experiences with others (Carstensen, Isaacowitz, and Charles 1999) was not supported. This may reflect that it is the quality and not quantity of relationships that matters in old age (Bruine de Bruin, Parker, and Strough 2020). Possibly, the coding captured more the number of somewhat fulfilling relationships than the quality of relationships overall. Older people may mention deeper but fewer relationships so that the communion score which is averaged across all life narrative segments is lower. However, our finding is consistent with results of Fritsch, Voltzenlogel, and Cuervo‐Lombard (2024) showing that narratives of self‐defining memories are less often about friendship in older adulthood than in younger and middle adulthood. In future studies, a more nuanced rating of overall communion (Adler et al. 2012) specifically in relationship memories might capture better a possible focus of emotional fulfillment in older age.

From reading the life narratives, we gained the impression that participants talked about topics that were somewhat in question or were still demanding narrative examination. Participants in their teen years tended to narrate their friendships with all ups and downs, and every romantic relationship they ever had was highlighted as important. We presume that relationships of older people are more continuous and stable and therefore discussed in less detail. Instead of narrating several single relationship episodes, the entire relationship experience might be summarized, as in this quote from an older female participant: “I am very grateful to my husband that he always stood behind our family and always takes very good care of our family and of me.”

The paucity of unfulfilled relative to fulfilled themes corresponds to the overall more positive than negative emotional tone in life narratives (Martin et al. 2023). Perhaps it is more difficult to talk about unfulfilled wishes, to admit to oneself and others that there is something missing in life, than talking about what has come true. Also, unfulfilled themes fit less into what is expected to be a proper life, as can be seen from the positivity of the cultural life script (Berntsen and Rubin 2004).

Fulfilled and unfulfilled themes varied independently from each other. Apparently, people who narrate their success or self‐mastery moments do not necessarily also talk about their failures, their loss of status or control. Likewise, people who share experiences of love and friendship do not automatically also talk about unfulfilled wishes of love and belonging. There appears to be no value‐independent, overall individual importance of agency or communion. Rather people seem to speak about fulfilled and about unfulfilled motives depending on what happened in life but also on the individual tendency to interpret life events in a more positive or negative way.

Girls and women narrated their lives with more communion than boys and men. This is in line with findings that girls' and women's autobiographical narratives focus more on other people (see for a review: Grysman and Hudson 2013) as well as with gender differences in the implicit affiliation motive (Drescher and Schultheiss 2016) and in communion in self‐descriptions (Diehl, Owen, and Youngblade 2004). We add to the literature by observing that this gender difference regards both fulfilled and unfulfilled communion, which might indicate that gender differences in motives do not depend on whether they are fulfilled or not.

Furthermore, consistent with earlier narrative findings in adults, life narratives did not differ in agency between genders. This deviation from normative, stereotypical, and self‐ reported gender differences in agency has not been discussed in the literature, maybe due to the yet limited findings. Given the absent to low correlation between self‐report and narrative measures of agency and communion (Grossbaum and Bates 2002; Grysman et al. 2016), this might reflect the more indirect, implicit nature of narrative content compared to explicit judgments about oneself. We might speculate that possibly, especially in younger cohorts, women construct their lives as agentively as their male counterparts, but that their conscious self‐concept lags behind.

### 3.1 Limitations and Strengths

The specific strengths of the study are its theoretical foundation, the life span sample, the longitudinal design, and the use of entire life narratives. One of the limitations is the relative homogeneity of our sample in terms of education, class, and culture. In future research, it will be important to compare these findings to samples from other social classes and other cultures. Studies suggest that the development of an increasingly self‐determined, agentic life narrative stems from conceptions of the good life that are specific to middle‐class individuals in Western countries (cf. Hatiboğlu Altunnar and Habermas 2018). Another limitation is that we cannot exclude cohort effects because we only had one cohort for each of the older age groups. Thus, we cannot exclude that the oldest cohort would have told less agentic and communal life narratives than the younger cohorts even at earlier stages of their lives.

Finally, the low to moderate rank‐order stability leaves plenty of room for individual influences on how agentic and communal a story is told. We had assumed that age‐related change in motivational themes is related to changes in motives and goals, which in turn are related to age‐graded life events. However, we did not test the influence of age‐related life events on motives and goals, nor that of motives and goals on themes of agency and communion in life narratives. Unfortunately, to systematically test the direct influence of life events such as marriage or transition to parenthood on mean‐level change, we had too few participants who experienced such events during the course of the study. Also, situational factors such as interviewer's personality and current mood might be considered.

When Yasemin grew older she repeated her narrative of coming to Germany, being an outsider, then changing her style and becoming a self‐confident person. At age 24, she added how she had gone to university and had joined political groups. At age 28, she recounted experiences of unity and empowerment when she engaged in student protests. Another 4 years later, she added how loved she had felt when she got married. Despite all change, her striving for agency and her need to belong stayed the central themes of her life story.

## Author Contributions

The study was conceptualized by N.F.K., who also performed the statistical analyses, and wrote the first draft of the manuscript. T.M. refined the statistical methodology and reviewed all data interpretations. L.C. contributed to the study's conceptual development and preliminary data exploration. F.S. reviewed and supervised the statistical approaches used in the study, while T.H. sourced the data and supervised the project at all stages. All authors discussed the findings, contributed to manuscript editing, and approved the final version.

## Ethics Statement

The current study utilized data from waves 1 through 4 of the MainLife study. Waves 3 and 4 were approved by the Ethics Committee of the Department of Psychology, Goethe University Frankfurt (2010 V01R1, 2014‐120). This study was not preregistered.

## Conflicts of Interest

The authors declare no conflicts of interest.

## Supporting information


Data S1.

Table S2.

Table S3.

Table S4.

## Data Availability

Coding manual, quantitative data, and analysis code have been made publicly available at the OSF and can be assessed at https://osf.io/a5drt/?view_only=44bf9ef8f5ca492cb6dc6f52d63062ee. To protect participants' confidentiality, we do not publish narrative data.

## References

[jopy12990-bib-0001] Adler, J. M. , E. D. Chin , A. P. Kolisetty , and T. F. Oltmanns . 2012. “The Distinguishing Characteristics of Narrative Identity in Adults With Features of Borderline Personality Disorder: An Empirical Investigation.” Journal of Personality Disorders 26, no. 4: 498–512. 10.1521/pedi.2012.26.4.498. 22867502 PMC3434277

[jopy12990-bib-0002] Bakan, D. 1966. The Duality of Human Existence: An Essay on Psychology and Religion. Chicago, IL: Rand Mcnally.

[jopy12990-bib-0003] Bar‐Yam Hassan, A. B. Y. , and M. Bar‐Yam . 1987. “Interpersonal Development Across the Life Span: Communion and Its Interaction With Agency in Psychosocial Development.” Contributions to Human Development 18: 102–128.

[jopy12990-bib-0004] Bates, D. , M. Maechler , B. Bolker , and S. Walker . 2015. “Fitting Linear Mixed‐Effects Models Using lme4.” Journal of Statistical Software 67, no. 1: 1–48. 10.18637/jss.v067.i01.

[jopy12990-bib-0005] Berntsen, D. , and D. C. Rubin . 2004. “Cultural Life Scripts Structure Recall From Autobiographical Memory.” Memory & Cognition 32, no. 3: 427–442. 10.3758/BF03195836. 15285126

[jopy12990-bib-0006] Bluck, S. , and T. Habermas . 2000. “The Life Story Schema.” Motivation and Emotion 24: 121–147. 10.1023/A:1005615331901.

[jopy12990-bib-0007] Bruine de Bruin, W. , A. M. Parker , and J. Strough . 2020. “Age Differences in Reported Social Networks and Well‐Being.” Psychology and Aging 35, no. 2: 159–168. 10.1037/pag0000415. 31697096 PMC7122684

[jopy12990-bib-0008] Buehler, J. L. , R. Weidmann , and A. Grob . 2021. “The Actor, Agent, and Author Across the Life Span: Interrelations Between Personality Traits, Life Goals, and Life Narratives in an Age‐Heterogeneous Sample.” European Journal of Personality 35, no. 2: 168–196. 10.1002/per.2275.

[jopy12990-bib-0009] Carstensen, L. L. , D. M. Isaacowitz , and S. T. Charles . 1999. “Taking Time Seriously: A Theory of Socioemotional Selectivity.” American Psychologist 54, no. 3: 165–181. 10.1037/0003-066X.54.3.165. 10199217

[jopy12990-bib-0010] Chodorow, N. 1978. The Reproduction of Mothering: Psychoanalysis and the Sociology of Gender. Berkeley and Los Angeles, CA: University of California Press.

[jopy12990-bib-0011] Conway, M. A. 2005. “Memory and the Self.” Journal of Memory and Language 53, no. 4: 594–628. 10.1016/j.jml.2005.08.005.

[jopy12990-bib-0012] Conway, M. A. , and A. Holmes . 2004. “Psychosocial Stages and the Accessibility of Autobiographical Memories Across the Life Cycle.” Journal of Personality 72, no. 3: 461–480. 10.1111/j.0022-3506.2004.00269.x. 15102035

[jopy12990-bib-0013] Conway, M. A. , L. V. Justice , and A. D'Argembeau . 2019. “The Self‐Memory System Revisited.” In The Organization and Structure of Autobiographical Memory, edited by J. Mace , 28–51. New York, NY: Oxford University Press.

[jopy12990-bib-0014] Conway, M. A. , and C. W. Pleydell‐Pearce . 2000. “The Construction of Autobiographical Memories in the Self‐Memory System.” Psychological Review 107, no. 2: 261–288. 10.1037/0033-295X.107.2.261. 10789197

[jopy12990-bib-0015] Craik, F. I. M. , and R. S. Lockhart . 1972. “Levels of Processing: A Framework for Memory Research.” Journal of Verbal Learning and Verbal Behavior 11: 671–684. 10.1016/S0022-5371(72)80001-X.

[jopy12990-bib-0016] Diehl, M. , S. K. Owen , and L. M. Youngblade . 2004. “Agency and Communion Attributes in adults' Spontaneous Self‐Representations.” International Journal of Behavioral Development 28, no. 1: 1–15. 10.1080/01650250344000226. 18592013 PMC2441921

[jopy12990-bib-0017] Drescher, A. , and O. C. Schultheiss . 2016. “Meta‐Analytic Evidence for Higher Implicit Affiliation and Intimacy Motivation Scores in Women, Compared to Men.” Journal of Research in Personality 64: 1–10. 10.1016/j.jrp.2016.06.019.

[jopy12990-bib-0018] Erikson, E. H. 1950. Childhood and Society. New York, NY: Norton & Co.

[jopy12990-bib-0019] Erikson, E. H. 1968. Identity: Youth and Crisis. New York, NY: Norton & Co.

[jopy12990-bib-0020] Fritsch, A. , V. Voltzenlogel , and C. Cuervo‐Lombard . 2024. “A Cross‐Sectional Study Using Self‐Defining Memories to Explore Personal Identity Throughout Adulthood.” Developmental Psychology 60, no. 2: 363–375. 10.1037/dev0001611. 38095997

[jopy12990-bib-0021] Gillespie, B. J. , J. Lever , D. Frederick , and T. Royce . 2015. “Close Adult Friendships, Gender, and the Life Cycle.” Journal of Social and Personal Relationships 32, no. 6: 709–736. 10.1177/0265407514546977.

[jopy12990-bib-0022] Gilligan, C. 1982. In a Different Voice: Psychological Theory and women's Development. Cambridge, MA: Harvard University Press.

[jopy12990-bib-0023] Grossbaum, M. F. , and G. W. Bates . 2002. “Correlates of Psychological Well‐Being at Midlife: The Role of Generativity, Agency and Communion, and Narrative Themes.” International Journal of Behavioral Development 26, no. 2: 120–127. 10.1080/01650250042000654.

[jopy12990-bib-0024] Grysman, A. , R. Fivush , N. A. Merrill , and M. Graci . 2016. “The Influence of Gender and Gender Typicality on Autobiographical Memory Across Event Types and Age Groups.” Memory & Cognition 44: 856–868. 10.3758/s13421-016-0610-2. 27068433

[jopy12990-bib-0025] Grysman, A. , and J. A. Hudson . 2013. “Gender Differences in Autobiographical Memory: Developmental and Methodological Considerations.” Developmental Review 33, no. 3: 239–272. 10.1016/j.dr.2013.07.004.

[jopy12990-bib-0026] Habermas, T. 2022. “The Longitudinal Study of Brief Life Narratives: Mainlife Study (2002‐2019). Study Report.” 10.26092/ELIB/1651.

[jopy12990-bib-0027] Habermas, T. , and S. Bluck . 2000. “Getting a Life: The Development of the Life Story in Adolescence.” Psychological Bulletin 126: 748–769. 10.1037/0033-2909.126.5.748. 10989622

[jopy12990-bib-0028] Habermas, T. , and C. de Silveira . 2008. “The Development of Global Coherence in Life Narratives Across Adolescence: Temporal, Causal, and Thematic Aspects.” Developmental Psychology 44: 707–721. 10.1037/0012-1649.44.3.707. 18473638

[jopy12990-bib-0029] Habermas, T. , and V. Diel . 2013. “The Episodicity of Verbal Reports of Personally Significant Autobiographical Memories: Vividness Correlates With Narrative Text Quality More Than With Detailedness or Memory Specificity.” Frontiers in Behavioral Neuroscience 7: 110. 10.3389/fnbeh.2013.00110. 23966918 PMC3746456

[jopy12990-bib-0030] Hagestad, G. O. , and P. A. Dykstra . 2016. “Structuration of the Life Course: Some Neglected Aspects.” In Handbook of the Life Course, edited by M. J. Shanahan , J. T. Mortimer , and M. K. Johnson , vol. II, 131–157. Cham, Switzerland: Springer. 10.1007/978-3-319-20880-0_6.

[jopy12990-bib-0031] Hatiboğlu Altunnar, N. , and T. Habermas . 2018. “Life Narratives Are More Other‐Centered, More Negative, and Less Coherent in Turkey Than in Germany: Comparing Provincial‐Turkish, Metropolitan‐Turkish, Turkish‐German, and Native German Educated Young Adults.” Frontiers in Psychology 9: 2466. 10.3389/fpsyg.2018.02466. 30581403 PMC6292932

[jopy12990-bib-0032] Havighurst, R. J. 1972. Developmental Tasks and Education. New York, NY: McKay.

[jopy12990-bib-0033] Heckhausen, J. 2018. “The Motivation of Developmental Regulation.” In Motivation and Action, edited by J. Heckhausen and H. Heckhausen , 745–782. Cham, Switzerland: Springer. 10.1007/978-3-319-65094-4_17.

[jopy12990-bib-0034] Heckhausen, J. , and R. Schulz . 1995. “A Life‐Span Theory of Control.” Psychological Review 102: 284–304. 10.1037/0033-295X.102.2.284. 7740091

[jopy12990-bib-0035] Holm, T. , D. K. Thomsen , and V. Bliksted . 2018. “Themes of Unfulfilled Agency and Communion in Life Stories of Patients With Schizophrenia.” Psychiatry Research 269: 772–778. 10.1016/j.psychres.2018.08.116. 30380593

[jopy12990-bib-0036] Hsu, N. , K. L. Badura , D. A. Newman , and M. E. P. Speach . 2021. “Gender, “masculinity,” and “femininity”: A meta‐analytic review of gender differences in agency and communion.” Psychological Bulletin 147, no. 10: 987–1011. 10.1037/bul0000343.

[jopy12990-bib-0037] Jensen, R. A. A. , D. K. Thomsen , M. Lind , N. Ladegaard , and V. F. Bliksted . 2021. “Storying the Past and the Future: Agency and Communion Themes Among Individuals With Schizophrenia and Depression.” Journal of Nervous and Mental Disease 209, no. 5: 343–352. 10.1097/NMD.0000000000001302. 33835953

[jopy12990-bib-0038] Köber, C. , and T. Habermas . 2017. “How Stable Is the Personal Past? Stability of Most Important Autobiographical Memories and Life Narratives Across Eight Years in a Life Span Sample.” Journal of Personality and Social Psychology 113, no. 4: 608–626. 10.1037/pspp0000145. 28333472

[jopy12990-bib-0039] Köber, C. , F. Schmiedek , and T. Habermas . 2015. “Characterizing Life Span Development of Three Aspects of Coherence in Life Narratives: A Cohort‐Sequential Study.” Developmental Psychology 51, no. 2: 260–275. 10.1037/a0038668. 25621758

[jopy12990-bib-0040] Martin, T. , N. F. Kemper , F. Schmiedek , and T. Habermas . 2023. “Lifespan Effects of Current Age and of Age at the Time of Remembered Events on the Affective Tone of Life Narrative Memories: Early Adolescence and Older Age Are More Negative.” Memory & Cognition 51: 1265–1286. 10.3758/s13421-023-01401-x. 36813991 PMC10368566

[jopy12990-bib-0041] McAdams, D. P. 1982. “Experiences of Intimacy and Power: Relationships Between Social Motives and Autobiographical Memory.” Journal of Personality and Social Psychology 42, no. 2: 292–302. 10.1037/0022-3514.42.2.292.

[jopy12990-bib-0042] McAdams, D. P. 1993. The Stories We Live by: Personal Myths and the Making of the Self. New York, NY: Guilford Press.

[jopy12990-bib-0043] McAdams, D. P. 1997. “Guided Autobiography.” https://www.sesp.northwestern.edu/docs/guided_autobiograph.pdf.

[jopy12990-bib-0044] McAdams, D. P. 2001. Coding Autobiographical Episodes for Themes of Agency and Communion. Illinois: Northwestern University Evanston.

[jopy12990-bib-0045] McAdams, D. P. , N. A. Anyidoho , C. Brown , Y. T. Huang , B. Kaplan , and M. A. Machado . 2004. “Traits and Stories: Links Between Dispositional and Narrative Features of Personality.” Journal of Personality 72, no. 4: 761–784. 10.1111/j.0022-3506.2004.00279.x. 15210016

[jopy12990-bib-0046] McAdams, D. P. , J. J. Bauer , A. R. Sakaeda , et al. 2006. “Continuity and Change in the Life Story: A Longitudinal Study of Autobiographical Memories in Emerging Adulthood.” Journal of Personality 74, no. 5: 1371–1400. 10.1111/j.1467-6494.2006.00412.x. 16958706

[jopy12990-bib-0047] McAdams, D. P. , B. J. Hoffman , E. D. Mansfield , and R. Day . 1996. “Themes of Agency and Communion in Significant Autobiographical Scenes.” Journal of Personality 64, no. 2: 339–377. 10.1111/j.1467-6494.1996.tb00514.x.

[jopy12990-bib-0048] McCabe, A. , and K. T. Dinh . 2016. “Agency and Communion, Ineffectiveness and Alienation: Themes in the Life Stories of Latino and Southeast Asian Adolescents.” Imagination, Cognition and Personality 36, no. 2: 150–171. 10.1177/0276236616648648.

[jopy12990-bib-0049] McLean, K. C. , and A. V. Breen . 2009. “Processes and Content of Narrative Identity Development in Adolescence: Gender and Well‐Being.” Developmental Psychology 45, no. 3: 702–710. 10.1037/a0015207. 19413426

[jopy12990-bib-0050] Mirowsky, J. , and C. E. Ross . 2007. “Life Course Trajectories of Perceived Control and Their Relationship to Education.” American Journal of Sociology 112, no. 5: 1339–1382. 10.1086/511800.

[jopy12990-bib-0051] Nusser, L. , D. Zimprich , and T. Wolf . 2023. “Themes of Trust, Identity, Intimacy, and Generativity in Important Autobiographical Memories: Associations With Life Periods and Life Satisfaction.” Journal of Personality 91, no. 5: 1110–1122. 10.1111/jopy.12786. 36256457

[jopy12990-bib-0052] Peters, I. , N. F. Kemper , F. Schmiedek , and T. Habermas . 2023. “Individual Differences in Revising the Life Story – Personality and Event Characteristics Influence Change in the Autobiographical Meaning of Life Events.” Journal of Personality 91, no. 5: 1207–1222. 10.1111/jopy.12793. 36415918

[jopy12990-bib-0053] Peters, I. , F. Schmiedek , and T. Habermas . 2024. “Narrating Lives Across 16 Years – Developmental Trajectories of Coherence and Relations to Well‐Being in a Life Span Sample.” Developmental Psychology. 10.1037/dev0001775 39250297

[jopy12990-bib-0054] Ross, C. E. , and J. Mirowsky . 2002. “Age and the Gender Gap in the Sense of Personal Control.” Social Psychology Quarterly 65, no. 2: 125–145. 10.2307/3090097.

[jopy12990-bib-0055] Sengsavang, S. , M. W. Pratt , S. Alisat , and P. Sadler . 2017. “The Life Story From Age 26 to 32: Rank‐Order Stability and Mean‐Level Change.” Journal of Personality 86: 788–802. 10.1111/jopy.12356. 29023734

[jopy12990-bib-0056] Wickham, H. 2016. Ggplot2: Elegant Graphics for Data Analysis. Cham, Switzerland: Springer. 10.1007/978-3-319-24277-4.

[jopy12990-bib-0057] Wiggins, J. S. 1991. “Agency and Communion as Conceptual Coordinates for the Understanding and Measurement of Interpersonal Behavior.” In Thinking Clearly About Psychology: Essays in Honor of Paul E. Meehl, Vol. 2. Personality and Psychopathology, edited by D. Cicchetti and W. M. Grove , 89–113. Minneapolis, MN: University of Minnesota Press.

[jopy12990-bib-0058] Woike, B. A. 2008. “A Functional Framework for the Influence of Implicit and Explicit Motives on Autobiographical Memory.” Personality and Social Psychology Review 12, no. 2: 99–117. 10.1177/1088868308315701. 18453474

[jopy12990-bib-0059] Wood, W. , and A. H. Eagly . 2012. “Biosocial Construction of Sex Differences and Similarities in Behavior.” Advances in Experimental Social Psychology 46: 55–123. 10.1016/B978-0-12-394281-4.00002-7.

