# Supplementary material for: Agency and Communion in Brief Entire Life Narratives Across the Life Span
Source: J Pers. 2024 Nov 9;93(5):1042–54. doi: 10.1111/jopy.12990 (PMC12421715; doi:10.1111/jopy.12990)
Supplement: Supplementary file 1 — Data S1. [file JOPY-93-1042-s001.docx]

**Supplemental Material**

**S1 Tests regarding gender, cohort, education, and dropouts**

The gender distribution of the cohorts did not differ significantly, X^2^ (1, N = 172) = .909, p = .970, *V* = .022.

Dropouts: We compared those participants who had dropped out by the fourth wave with those who had not. They did neither differed by gender X^2^ (1, N = 172) = 1.016, p = .314, *V* = .223, nor by cohort X^2^ (1, N = 172) = 8.539, p = .129, *V* = .017. The four younger cohorts were homogenous as to education, and in the two older cohorts dropouts did not differ from others in years of school, *F*(1, 56) = .985, *p* = .325, η^2 =^ .022.
