# Supplementary material for: Agency and Communion in Brief Entire Life Narratives Across the Life Span
Source: J Pers. 2024 Nov 9;93(5):1042–54. doi: 10.1111/jopy.12990 (PMC12421715; doi:10.1111/jopy.12990)
Supplement: Supplementary file 2 — Table S2. [file JOPY-93-1042-s002.docx]

| **Table S2**  *Categories and Coding Examples of Agency* | | |
| --- | --- | --- |
| Code | Definition | Example |
| Self-Mastery | The protagonist strives successfully to master, control, enlarge, or perfect the self. Through action, thought, or experience, the person becomes a more powerful agent in the world. | “And at some point the dream arose, I want to become a sailor, [...] and I really went through with it.” |
| *Unfulfilled* | The protagonist strives unsuccessfully to strengthen the self; is at the mercy of circumstances; can not initiate change. | “For years I couldn't gather myself, I was always so very quiet and never dared to say anything.” |
| Status/ Victory | The protagonist attains heightened status, position, or prestige; seeks recognition or honors from others, especially in competitive situations. | “I earned so much money in the meantime [...] my wife never had to go to work, I bought my son a car for his high school graduation.” |
| *Unfulfilled* | The protagonist tries in vain to attain heightened status, position, or prestige; feels belittled. | “The teacher who had me in the class thought I was mentally deficient and said I had to repeat the class.” |
| Achievement/ Responsibility | The protagonist has substantial success in the achievement of tasks, jobs, or instrumental goals or in the assumption of important responsibilities. | “We also played soccer, which I am also very good at because I am in the club.” |
| *Unfulfilled* | The protagonist has no success in the achievement of tasks, jobs, or instrumental goals or in the assumption of important responsibilities. | “We used to do dictations and I always got bad grades there, even though I always practiced really hard.” |
| Empowerment | The protagonist is enlarged, enhanced, empowered through their association with an especially powerful source or an influential person. | “My guardian angel helped me overcome this crisis as well.” |
| *Unfulfilled* | The protagonist strives in vain for the support or guidance of an empowering force or an influential person. | *Not coded* |
