# Supplementary material for: Agency and Communion in Brief Entire Life Narratives Across the Life Span
Source: J Pers. 2024 Nov 9;93(5):1042–54. doi: 10.1111/jopy.12990 (PMC12421715; doi:10.1111/jopy.12990)
Supplement: Supplementary file 3 — Table S3. [file JOPY-93-1042-s004.docx]

| **Table S3**  *Categories and Coding Examples of Communion* | | |
| --- | --- | --- |
| Code | Definition | Example |
| Love/Friendship | The protagonist experiences an enhancement of erotic love or friendship toward another person. | “Alright, and so, again, a really great and intimate relationship developed from that weekend.” |
| *Unfulfilled* | The protagonist has negative feelings about lack of love or friendship or the end of a relationship. | “A year and a half later, she then – well we're not together anymore and that's actually quite hard for me.” |
| Dialogue | The protagonist experiences a reciprocal and noninstrumental form of communication or dialogue with another person or group of others. | “[...] and we had long conversations and very deep and we both cried a lot.” |
| *Unfulfilled* | The protagonist has negative feelings about the lack of meaningful communication. | “My two brothers are very simple people [...] and so we can't talk about such deeper things either.” |
| Caring/ Help | The protagonist provides care, assistance, nurturance, aid, support, or therapy for another, providing for the welfare or well-being of the other. | “[…] and even when I'm not there, I think about her a lot and worry when I realize that something is wrong.” |
| *Unfulfilled* | The protagonist strives unsuccessfully to provide care, assistance, nurturance, aid, support, or therapy for another. | “[...] and dragged me down, I couldn't help him either, he didn't let me help him.” |
| Unity/ Togetherness | The protagonist experiences a sense of oneness, unity, harmony, togetherness, belongingness, or solidarity with a group of people, a community, or even all of humankind. | “So a year later we were really a cooperating class, we all liked each other and it was a really good group.” |
| *Unfulfilled* | The protagonist has negative feelings about lack of unity, harmony, belonging, and solidarity with a group; feels isolated or abandoned. | “I was always the outsider and, I don't know, nobody liked me.” |
