# Supplementary material for: Agency and Communion in Brief Entire Life Narratives Across the Life Span
Source: J Pers. 2024 Nov 9;93(5):1042–54. doi: 10.1111/jopy.12990 (PMC12421715; doi:10.1111/jopy.12990)
Supplement: Supplementary file 4 — Table S4. [file JOPY-93-1042-s003.docx]

**Table S4**

*Stability by Two Cohorts Each*

|  | Agency | Communion | Unfulfilled Agency | Unfulfilled Communion | *n* |
| --- | --- | --- | --- | --- | --- |
| Compared Time Interval | |  |  |  |  |
| Cohorts 1, 2  *2 Weeks*  Wave 1  *4 Years* | .43 | .62 | .31 | .37 | 49 |
| Wave 1-2 | .36 | .22 | .07 | -.03 | 52 |
| Wave 2-3 | .21 | .38 | .12 | .24 | 47 |
| Wave 3-4 | .52 | .33 | .29 | .35 | 44 |
| *8 Years* |  |  |  |  |  |
| Wave 1-3 | .08 | .18 | .16 | .03 | 50 |
| Wave 2-4 | .22 | .51 | -.10 | .00 | 43 |
| *12 Years* |  |  |  |  |  |
| Wave 1-4 | .02 | .17 | -.07 | .29 | 46 |
| Cohorts 3, 4  *2 Weeks*  Wave 1  *4 Years* | .48 | .51 | .54 | .38 | 52 |
| Wave 1-2 | .11 | .57 | .40 | .14 | 52 |
| Wave 2-3 | .40 | .42 | .19 | .37 | 47 |
| Wave 3-4 | .24 | .61 | .57 | .40 | 38 |
| *8 Years* |  |  |  |  |  |
| Wave 1-3 | .33 | .26 | .30 | .45 | 49 |
| Wave 2-4 | .09 | .54 | .39 | .23 | 40 |
| *12 Years* |  |  |  |  |  |
| Wave 1-4 | -.03 | .54 | .23 | .48 | 41 |
| Cohorts 5, 6  *2 Weeks*  Wave 1  *4 Years* | --- | --- | --- | --- | --- |
| Wave 1-2 | --- | --- | --- | --- | --- |
| Wave 2-3 | .35 | .33 | .31 | .33 | 51 |
| Wave 3-4 | .58 | .44 | .55 | .52 | 47 |
| *8 Years* |  |  |  |  |  |
| Wave 1-3 | --- | --- | --- | --- | --- |
| Wave 2-4 | .61 | .12 | .47 | .41 | 48 |
| *12 Years* |  |  |  |  |  |
| Wave 1-4 | --- | --- | --- | --- | --- |
|  |  |  |  |  |  |
